# Doppler ultrasonography in the diagnosis of Graves disease: A non-invasive, widely under-utilized diagnostic tool

**DOI:** 10.4103/0256-4947.55307

**Published:** 2009

**Authors:** Saleh Aldasouqi, Ahmad Sheikh, Pamela Klosterman

**Affiliations:** From the Department of Medicine, Saint Francis Medical Center, Missouri, USA

**To the Editor:** We present the case of a patient with Graves disease (GD) to draw attention to the practical reliability of ultrasound (US) Doppler in the diagnosis of GD. We believe that this simple, non-expensive, non-invasive, and portable tool is under-utilized in the diagnosis of GD, and that most clinicians rely heavily on radioiodine (RAI) uptake scan for this purpose. Besides the radiation exposure encountered in RAI scintigraphy and the length and multi-phase application of the procedure, RAI scan is (roughly) 5-10 times more expensive than US. The diagnosis of GD can often be based solely on clinical grounds and simple blood tests, and without the need for imaging studies. The majority of such patients are first seen by their family doctors, and by the time a referral is made to an endocrinologist, both US and RAI scans are (often) already done. The problem is that even in the case where a US is ordered, no efforts are made to emphasize the characteristic intense Doppler flow in these cases; this pattern, referred to as “inferno”, is almost pathognomonic for GD.^1^–^3^

**Figure 1:**
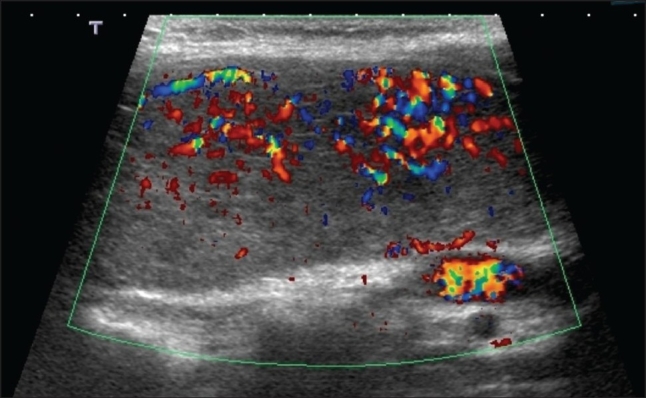
Ultrasound of the thyroid showing an intense vascularity on color Doppler that is consistent with an “inferno”.

A 28-year-old woman presented to the endocrine clinic with severe symptoms of hyperthyroidism of 2 months duration. She had no significant eye symptoms. On physical examination, she was found to have moderately severe hyperthyroidism, but she was clinically not in distress and was not in a thyroid storm. She had approximately 50 g symmetric firm goiter, with very loud bruits. Her thyroid function test showed thyroid stimulating hormone of <0.001 mIU/L; free T4 of 105.53 pmol/L; and total T3 >12.32 pmol/L. The patient brought an ultrasound image (Figure 1). As shown in the figure, the color Doppler depicts a high soft tissue vascularity, described as an “inferno”. We believe that this particular feature of the US–“the inferno”–is under-utilized in clinical practice. It is anecdotally observed that a lot of physicians consider an RAI scan as the mainstay imaging study for hyperthyroidism, and often do not get US if GD is confirmed by the isotope scan. Even if US is ordered in the diagnostic work up of GD, we noted from cases referred for consultation that Doppler is not utilized. Furthermore, we noted that the literature is relatively scarce on the role of US in the diagnosis of GD.

Ralls and associates first used color Doppler sonography in 1988 for the diagnosis of GD.^1^ They used the term “inferno” to refer to the pulsatile flow pattern in GD, which was obvious in all 16 patients with GD in their study, as compared to controls. Arslan and associates were able to demonstrate the peculiar diffuse hypervascularity in all 23 GD patients of their study.^2^ Cappelli et al recently published a large prospective study^3^ on hyperthyroid patients (n=1470, with 426 with GD), reporting excellent sensitivity and specificity of US in GD (by vascular Doppler pattern). The US Doppler had a 95% sensitivity and a 95% specificity, as compared to those of RAI scan (97% and 99%, respectively).

More recently, Kumar et al^4^ reported the excellent diagnostic accuracy of peak systolic velocity of the inferior thyroid artery as measured by US Doppler in hyperthyroidism. In a study of 65 patients with hyperthyroidism (GD versus thyroiditis), this particular technique had a sensitivity of 96% and specificity of 95%, with a peak systolic velocity of 40 cm/sec as a cut-point diagnostic value. This technique has thus added a quantitative tool to supplement the qualitative tool of tissue vascularity; the two tools can be very helpful in the diagnosis of GD.

However, Levine cautioned^5^ that cases of subacute thyroiditis would have any degree of vascularization (which would depend on the phase of the condition). Furthermore, no data are available on US findings in pregnant women with hyperemesis gravidara, which can be difficult to distinguish from GD in early pregnancy. Therefore, we believe that larger studies are warranted to evaluate the validity of US Doppler in the diagnosis of GD in pregnant women, especially in early gestation.

While GD can be often diagnosed with a certain degree of confidence by clinical evaluation and simple blood tests, some cases may be difficult to diagnose. This difficulty is especially encountered in the differentiation between GD and thyroiditis, and in particular in mild cases where pathognomonic features of GD, e.g. opthalmopathy, are absent. While RAI scan is the standard imaging study used in such situations, we believe that US may provide an alternative, especially when RAI scan is less available, less affordable (roughly $1500 vs $150, depending on geographical location), or contraindicated (e.g. pregnancy or lactation). However, clinicians should be aware of the occasional overlapping that may be encountered between GD and subacute thyroiditis.

It is prudent to point out that US should be considered in every patient with GD because incidental small thyroid cancers (<10 mm) could be missed by physical examination or RAI scans, as emphasized by Cappelli et al.^3^ In this regard, clinical examination for the detection of thyroid nodules in general has a low sensitivity and specificity.^6^–^8^ This is especially important in patients with goiters, such as in patients with GD where small nodules could be embedded within these goiters.^3^

We believe that radiology facilities should have an US protocol with Doppler patterns (color or power or both) in all patients referred for US, with a clinical diagnosis of GD, describing the pattern and whether it qualifies as an “inferno”. Reporting radiologists should then emphasize this in their reports. Furthermore, Doppler peak systolic velocity of inferior thyroidal artery seems to be similarly promising in the diagnosis of GD.
